# Genetic Variations and mRNA Expression of NRF2 in Parkinson's Disease

**DOI:** 10.1155/2017/4020198

**Published:** 2017-05-02

**Authors:** Caroline Ran, Karin Wirdefeldt, Lovisa Brodin, Mehrafarin Ramezani, Marie Westerlund, Fengqing Xiang, Anna Anvret, Thomas Willows, Olof Sydow, Anders Johansson, Dagmar Galter, Per Svenningsson, Andrea Carmine Belin

**Affiliations:** ^1^Department of Neuroscience, Karolinska Institutet, 17177 Stockholm, Sweden; ^2^Department of Clinical Neuroscience, Karolinska Institutet, 17177 Stockholm, Sweden; ^3^Department of Medical Epidemiology and Biostatistics, Karolinska Institutet, 17177 Stockholm, Sweden; ^4^Department of Neurobiology, Care Sciences and Society, Karolinska Institutet, 14183 Huddinge, Sweden; ^5^Department of Women's and Children's Health, Karolinska Institutet, 17176 Stockholm, Sweden

## Abstract

Nuclear factor erythroid 2-like 2* (NRF2)* encodes a transcription factor regulating mechanisms of cellular protection and is activated by oxidative stress.* NRF2* has therefore been hypothesized to confer protection against Parkinson's disease and so far an* NRF2* haplotype has been reported to decrease the risk of developing disease and delay disease onset. Also* NRF2* adopts a nuclear localization in Parkinson's disease, which is indicative of increased* NRF2* activity. We have investigated the association between* NRF2* and Parkinson's disease in a Swedish case-control material and whether* NRF2* expression levels correlate with* NRF2* genetic variants, disease, or disease onset. Using pyrosequencing, we genotyped one intronic and three promoter variants in 504 patients and 509 control subjects from Stockholm. Further, we quantified* NRF2* mRNA expression in EBV transfected human lymphocytes from patients and controls using quantitative real-time reverse transcription PCR. We found that one of the promoter variants, rs35652124, was associated with age of disease onset (Χ^2^ = 14.19, *p* value = 0.0067).* NRF2* mRNA expression levels however did not correlate with the rs35652124 genotype, Parkinson's disease, or age of onset in our material. More detailed studies on* NRF2* are needed in order to elucidate how this gene affects pathophysiology of Parkinson's disease.

## 1. Introduction

Parkinson's disease (PD) is the second most common neurodegenerative disorder in the aging population; approximately 20,000 people are affected by PD in Sweden which has a population around 9.6 million. PD is characterized by degeneration of dopamine (DA) neurons in substantia nigra (SN), but there is degeneration of other neurons as well. Although there is a relatively large body of knowledge on the pathology of PD, there is little understanding of the etiology. The existing treatment for PD today, for example, replacing DA, improving the effect of DA, or inhibiting the breakdown of DA, does not cure the disease but only reduces the symptoms. In order to develop better ways of combating PD or better still prevent clinical symptoms altogether, one must decipher the causes of disease.

Typically, candidate genes for PD do not specifically relate to the DA system. Instead, they tend to serve general cellular purposes, such as to protect against reactive molecules or participate in mitochondrial function [1]. One gene that has been suggested to confer protection against PD is the nuclear factor erythroid 2-like 2* (NRF2)* (OMIM 600492), on chromosome 2q31. It belongs to a family of genes together with* NFE2* (OMIM 601490) and* NFE2L1* (OMIM 163260), encoding basic leucine zipper (bZIP) transcription factors [2]. In normal conditions, NRF2 is not active; it resides in the cytoplasm bound to KEAP1 (kelch-like ECH association protein 1), a ubiquitin E3 ligase complex, which mediates NRF2 degradation by the proteasome. In response to oxidative stress, NRF2 is released from KEAP1 and translocated from the cytoplasm into the nucleus where it transactivates expression of genes with antioxidant activity, detoxifying enzymes, cell survival, and anti-inflammatory factors [3]. Expression of* Nrf2*, the rat orthologue of* NRF2*, decreases with increasing age and is accompanied by a drop in activity of phase II antioxidant enzymes, which are upregulated by* NRF2* [4–6]. Subsequently, the decrease in* NRF2* expression results in increased sensitivity to oxidative damage.

In PD, nuclear localization of* NRF2* has been reported to be strongly induced in nigral neurons, but this response may be insufficient to protect neurons from degeneration [7]. Five genetic association studies have been published on* NRF2* in relation to PD with variable results [8–12]. In 2010, von Otter et al. performed a genetic study in a Swedish and a Polish PD material and identified a haplotype including three promoter single nucleotide polymorphisms (SNPs) and five tag SNPs that was associated with decreased risk of PD as well as later age of onset of PD [11]. The association with delayed disease onset was later replicated in an extended study comprising more than 1000 PD patients [12]. Another replication study on a subset of these SNPs in a Taiwanese PD population could not confirm these findings [8]. The importance of* NRF2* SNPs has also been assessed in a Chinese PD population in which an association was discovered with two exonic SNPs but not with the promoter SNPs suggested by von Otter et al. [9, 11]. Yet another SNP was found to be associated with PD risk in an Australian sample and, in this study, several SNPs and haplotypes were also found to affect the age of onset [10]. What is more, haplotype association has also been reported between* NRF2* and Alzheimer's disease (AD) as well as with age-related cataract and amyotrophic lateral sclerosis (ALS) [13, 14]. Overall it seems likely that genetic variants in* NRF2* affect both the risk of developing neurodegenerative disorders such as PD and the age of onset, but as different sets of SNPs were included in these studies, as well as the differential distribution of haplotypes in populations with different ethnicity, it is difficult to pinpoint what combination of SNPs is responsible for the reported associations.

*NRF2* may play an important role in cellular protection in neurodegenerative diseases as well as being a viable therapeutic target in the future. As described above, there are several indications supporting the involvement of* NRF2* in neurodegenerative disorders. In the present study, we investigated four genetic variants in* NRF2*, rs35652124 (A>G), rs6706649 (G>A), rs6721961 (C>A), and rs2001350 (A>G), and their possible association with PD in our large homogenous Swedish case-control material in order to expand on the knowledge on* NRF2* genetic variations as a risk factor in PD. The SNPs included in our study have previously been reported to be associated with PD in a Caucasian material consisting of Swedish and Polish individuals [11]. We additionally wanted to examine whether the* NRF2* gene is differently expressed in patients and controls or affecting age of onset and if genetic variants identified to be associated with PD in this study affected expression in vivo.

## 2. Materials and Methods

### 2.1. Patients and Controls

PD patients were recruited to the study during routine outpatient visits to the Neurology clinic at Karolinska University Hospital, Stockholm, Sweden. Patients (*n* = 504) were unrelated of Swedish origin, mean age was 67.2 years, age at diagnosis was 59.4 years, and 36.7% were females (Table 1). All patients fulfilled the “UK Parkinson's Disease Society's Brain Bank Clinical Diagnostic Criteria” for idiopathic PD except that 28.2% reported one or more first, second, or third-degree relative diagnosed with PD [15]. Early onset (EO) was defined as being less than or equal to 50 years of age at diagnosis and late onset (LO) was defined as 51 years or more at diagnosis. As controls (*n* = 509) we included neurologically healthy individuals visiting the Neurology Clinic, spouses of PD patients, anonymous blood donors, and individuals from the SNAC-K project (The Swedish National Study on Aging and Care in Kungsholmen, http://www.snac-k.se/), mean age was 71.8 years, and 57.3% were females (Table 1). Samples from patients and controls were collected after informed consent and approval of the local ethics committee Stockholm. DNA was obtained from blood samples using standard protocols. Blood from patients and control individuals randomly selected from the individuals recruited at the Neurology Clinic was used for Epstein-Barr virus (EBV) transfection of B-lymphocytes, EO patients (*n* = 10), LO patients (*n* = 10), and controls (*n* = 10). All investigations on human subjects were carried out following the rules of the Declaration of Helsinki of 1975, revised in 2008. A subset of the patients (*n* = 330) and controls (*n* = 317) was previously genotyped in a preliminary study published in a doctoral thesis [16].

### 2.2. Genotyping by Pyrosequencing and qPCR

We genotyped four SNPs in* NRF2*: rs35652124, rs6706649, rs6721961 in the promoter, and rs2001350. Genotyping of the three promoter SNPs was performed by pyrosequencing [17]. Rs2001350 was genotyped by TaqMan® quantitative real-time PCR (qPCR). For pyrosequencing, a PCR was run using Taq polymerase enzyme and two oligonucleotide primers in order to isolate a sequence of 209 base pairs containing the three promoter SNPs; forward primer, 5′-GAATGGAGACACGTGGGAGT-3′; and reverse primer, 5′-ACTTTACCGCCCGAGAATG-3′ (Thermo Fisher Scientific, Hägersten, Sweden). The forward primer was biotinylated at the 5′ end. The PCR was programmed as follows: denaturation, at 95°C for 5 minutes, 45 cycles of amplification at 95°C for 20 sec, 57°C for 20 sec, and 72°C for 30 sec, and terminal elongation at 72°C for 7 minutes. The forward strand of the PCR product was captured and isolated on streptavidin coated sepharose beads and then purified according to manufacturer's instructions using a filter-probe vacuum prep-tool. The single stranded DNA fragment was incubated with a sequencing primer and annealed for 2 min at 80°C. SNPs rs35652124 and rs6706649 could be sequenced with the same sequence primer, 5′-TCACCCTGAACGC-3′, and rs6721961 was sequenced with sequencing primer 5′-GGAGATGTGGACA-3′. Sequences were analyzed using a pyrosequencing PSQ 96MA system (BIOTAGE AB, Uppsala, Sweden) using PyroMark Gold Q96 Reagents (QIAGEN Nordic, Sollentuna, Sweden). Rs2001350 was analyzed with TaqMan Fast 7500 qPCR. Rs2001350 was genotyped with a premade SNP genotyping assay, assay ID number C__11634985_10. We used a standard cycling program: pre-PCR reading at 60°C for one minute, polymerase activation at 95°C for 10 minutes, 55 cycles comprising denaturation at 92°C for 15 seconds and annealing/extension at 60°C for 1 minute, and last post-PCR reading at 60°C for 1 minute. TaqMan genotyping mix and assays were purchased from Applied Biosystems/Life Technologies (Life Technologies Europe BV, Stockholm, Sweden).

### 2.3. EBV Transfection of Lymphocytes

B-Lymphocytes were isolated from full blood using Ficoll-Paque according to manufacturer's instructions (GE Healthcare Bio-Sciences Corp., Piscataway, NJ, USA). Lymphocytes were cultivated in RPMI 1640 medium (SIGMA, St. Louis, MO, USA) with 20% fetal calf serum, L-glutamine (200 mM), penicillin-streptomycin (5000 *μ*g/mL) (Invitrogen, Carlsbad, CA, USA), and cyclosporine (1 *μ*L/mL, Apoteket, Stockholm, Sweden). Transfection was achieved by adding and filtering supernatant of Epstein-Barr virus (EBV) infected B95-8 cells to the medium. When immortalized, cells were frozen and kept at −140°C until use [18].

### 2.4. Reverse Transcription qPCR (qRT-PCR)

EBV transfected B-lymphocytes were thawed, reseeded, and harvested when cell count reached around 5 million cells and kept in −130°C. RNA was extracted from frozen cells using RNeasy Mini Kit according to manufacturer's instructions (QIAGEN Nordic, Sollentuna, Sweden). RNA concentration was measured and DNA contamination was eliminated. In order to avoid sample degradation, stable cDNA was synthesized from the RNA using a QuantiTect Reverse Transcription Kit (QIAGEN Nordic). The cDNA template was used for quantifying gene expression of* NRF2* in PD patients and controls by qRT-PCR. Gene specific primers were designed for exon four of* NRF2*, which is present in all mRNA transcripts: forward primer 5′-CTTTTGGCGCAGACATTCC-3′ and reverse primer 5′-AAGACTGGGCTCTCGATGTG-3′.* GAPDH* was used as housekeeping gene: forward primer 5′-AGCCACATCGCTCAGACA-3′ and reverse primer 5′-GCCCAATACGACCAAATCC-3′ (Thermo Fisher Scientific).* NRF2* primer pair gave an* R*^2^ value of 0.9507 and* GAPDH* primes gave an* R*^2^ value of 0.9809. The qRT-PCR reaction was performed according to standard protocols, using triplicates of each sample and SYBR green qRT-PCR mastermix from Applied Biosystems (Applied Biosystems, Life Technologies Europe BV, Stockholm, Sweden). The cycler was programmed as follows: holding stage for pre-PCR read and enzyme activation: 50°C for 2 min and 95°C for 10 min, amplification for 45 cycles: 95°C for 15 sec, 60°C for 1 min, and last melting curve and post-PCR read: 95°C for 15 sec, 60°C for 1 min, 95°C for 30 sec, and 60°C for 15 sec.

### 2.5. Statistical Analysis and Image Analysis

Genotype and allele frequencies were compared between PD patients and controls. Association for genotypes was evaluated with Chi square (*Χ*^2^) test and allele association was analyzed using Fisher's exact test. We used GraphPad Prism 5.03 (GraphPad Software Inc., La Jolla, CA, USA) for the analysis, significance level of 5% and two-sided *p* values. Bonferroni correction was used to correct for multiple testing. To control for the skewed gender distribution in the patient group (males = 63.3%), genotype analysis was verified using a logistic regression with gender as cofactor; analysis was run in PLINK v1.07 [19]. Hardy-Weinberg equilibrium was evaluated using a free online software through a *Χ*^2^ based test [20]. Haplotype analysis was run using the software Haploview4.2 [21]. qRT-PCR data was analyzed in qBase 1.3.3, a software used for automated analysis of real-time quantitative PCR data [22], and evaluated with a student's *t*-test or a one-way ANOVA using GraphPad Prism 5.03.

## 3. Results

### 3.1. Genotyping by Pyrosequencing and qPCR

We have genotyped four common SNPs in the* NRF2* gene in 504 PD patients from the Stockholm area of Sweden and 509 geographically matched control individuals. One genetic variation, rs2001350, lies in the large second intron of the gene; the three other SNPs, rs35652124, rs6706649, and rs6721961, are promoter SNPs. Results for the genotype and allele analysis can be found in Table 2. Genotype and allele association analysis revealed no significant associations with PD. All four SNPs were in Hardy-Weinberg equilibrium (data not shown). The logistic regression, which included gender as a covariate, was run under a genotypic model with 2 degrees of freedom confirming the results from the genotype association analysis (supplementary table 1 in Supplementary Material available online at https://doi.org/10.1155/2017/4020198), verifying that there was no bias introduced by the skewed gender distributions in the patient and control group. In order to analyze the combined effect of several SNPs in* NRF2*, we further ran a haplotype analysis, which did not reveal any association between these four SNPs and PD (supplementary table 2).

When patients were stratified in groups of EO or LO, genotype association analysis showed that one of the promoter SNPs, rs35652124, was significantly associated with EO PD when compared to controls (*Χ*^2^ = 14.19 *p* = 0.0067), (Table 3); both the WT genotype AA and the mutated genotype GG of rs35652124 were observed more frequently in PD patients with EO than in individuals in the two other groups, while EO heterozygous carriers were significantly fewer. The significance remained after correction for multiple testing, *p* = 0.027. The association was dissected further, which showed that the significance was dependent on the group of patients with early onset. The significance remained when comparing controls to EO patients only (*p* = 0.019) and when comparing LO patients to EO patients (*p* = 0.0013), but not when comparing controls to LO patients (*p* = 0.20). An additional analysis comparing patients with EO PD against LO PD and controls under a recessive model (AA + GA versus GG) and a dominant model (AA versus GA + GG), respectively, confirmed the association under a dominant model (*Χ*^2^ = 6.68, *p* = 0.013) and enabled us to establish the causality between the absence of G alleles and EO PD. Allele frequencies for rs35652124 were not associated with age at diagnosis (*p* = 0.15), nor did genotype or allele for rs6706649, rs6721961, and rs200350 (supplementary table 3).

### 3.2. mRNA Expression Analysis

**Figure 1:**
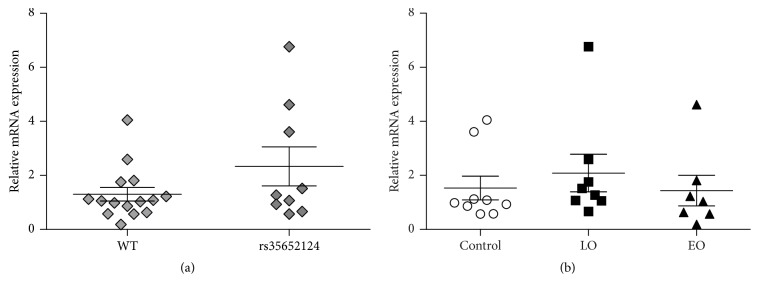
*rs35652124 genotype and age of onset do not correlate with NRF2 mRNA expression*.* NRF2* mRNA expression in EBV transfected human lymphocytes. Values were normalized to* GAPDH* mRNA and to a reference sample consisting of cDNA from all control individuals; groups were compared using a *t*-test (a) or one-way ANOVA (b) analysis. Error bars: standard error of mean. In (a) mRNA expression of all subjects was divided into two groups depending on their genotype for the promoter SNPs rs35652124. WT: subjects wild type for rs35652124 and rs35652124; subjects heterozygous (*n* = 8) and homozygous (*n* = 1) for rs35652124, *p* = 0.121. In (b) mRNA levels are analyzed according to disease status; LO: late onset PD; EO: early onset PD; *p* = 0.695.

**Figure 2:**
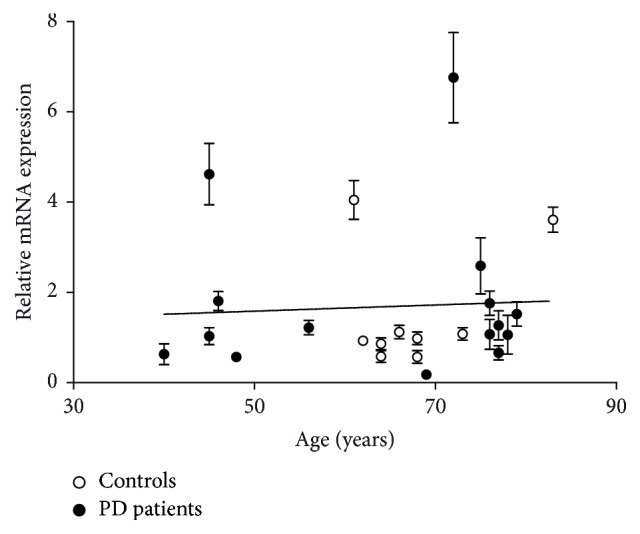
*mRNA levels do not correlate with age*.* NRF2* mRNA expression in EBV transfected lymphocytes from individuals of different age spanning from 40 years to 80; PD patients are represented in black and controls in white. Each data point corresponds to the mean value of triplicates normalized to* GAPDH* and to a reference sample consisting of cDNA from all control individuals; error bars: standard error of mean. Correlation between age and expression of* NRF2* was evaluated using linear regression and was found nonsignificant (slope = 0.0068, *p* = 0.799) when the entire material was analyzed regardless of disease and when controls were analyzed separately (regression line not shown, slope = 0.060, *p* = 0.429).

Since promoter SNPs in* NRF2* have been suggested to influence gene expression in vitro, we further wanted to investigate whether gene expression correlated with the rs35652124 genotype [23]. We performed qRT-PCR on a subset of the patients included in our genetic study; mRNA levels were normalized to* GAPDH* expression and to a reference sample consisting of mixed cDNA from all control individuals. mRNA expression in wild-type (WT) carriers of the promoter SNP rs35652124 (*n* = 15) was compared to individuals carrying one (*n* = 8) or two (*n* = 1) mutated alleles at this position. We found no significant difference between the carriers and noncarriers, (Student's *t*-test, *p* = 0.121) (Figure 1(a)). It is noteworthy that the individual with the highest relative* NRF2* expression (6.76) was homozygous GG for rs35652124 but carried the WT allele of the three other SNPs; exact mRNA levels and genotypes are found in supplementary table 4. We additionally compared* NRF2* mRNA levels in healthy controls (*n* = 9) and PD patients (*n* = 15) and found no significant difference (*p* = 0.712, supplementary table 4). Also when stratifying the material into two patient groups with LO PD (*n* = 8) or EO PD (*n* = 7) expression levels were similar in all three groups (*p* = 0.695) (Figure 1(b), supplementary table 4).* NRF2* mRNA levels in these individuals were quite variable, displaying a few higher expressing individuals in all groups. Last we analyzed the expression data with respect to age using linear regression (Figure 2, supplementary table 4). We found no correlation between expression levels of* NRF2* and age either when analyzing the entire sample regardless of disease status (*p* = 0.799) or in controls alone (*p* = 0.429).

## 4. Discussion

We have found a genotype association of rs35652124 with EO PD. Genotype frequencies for this SNP were difficult to interpret, as the EO patients were more often homozygous for both the wild-type and mutant allele. This could possibly mean that the mode of penetrance of this genetic variation is unlikely to be additive or multiplicative. When combining the genotypes, we found that both genotypes comprising the mutated allele, heterozygous GA or homozygous GG, were more common in the control group and PD patients with late onset, which is consistent with the minor allele G being rarer in the early onset population. Analyzing the material under a dominant model confirmed the association between the absence of G alleles and earlier disease onset. We therefore stipulate that the G allele confers some protection against PD by delaying the onset of symptoms. von Otter et al. previously reported a promoter haplotype consisting of the wild-type alleles of rs35652124, rs6706649, and rs6721961 associated with later age of onset and less risk of developing disease [11]. Together, these datasets point to a disease modifying role for promoter SNPs, in particular rs35652124.

In vitro data indicate that the promoter SNPs analyzed by us could influence the transcriptional activity of* NRF2* [23, 24]. The mutated allele of rs6706649 and rs6721961 has been reported to cause a drastic decrease of* NRF2* gene transcription in transfected cells, while decrease is less pronounced when the mutated allele of rs35652124 is combined with the mutated allele of rs6706649. This information, together with the results from our genetic analysis, prompted us to investigate the functional implication of the rs35652421 SNP in human tissue [23, 24].* NRF2* mRNA expression was quantified using qRT-PCR in patient specific cell-lines. Although qRT-PCR analysis showed no significant differences in mRNA levels when subjects were stratified according to genotype, quantification also revealed that* NRF2* expression levels were quite variable in both groups, and the standard error of mean in these groups was consequently large. Further analysis of gene expression data did not reveal any difference between control individuals and PD patients or between groups of patients receiving a diagnosis before and after the age of 50 years. There was still great variability between individuals within all groups. Furthermore, the variable expression levels did not correlate with the age of the study participants (spanning from 40 to 83 years), which is surprising considering previous reports of Nrf2 decreasing with increasing age [4, 6]. The marked divergence in expression levels within the different groups in our study indicates that there is a regulatory parameter not included in our analysis. We might therefore hypothesize that there are more regulatory* NRF2* SNPs which could influence the gene expression of* NRF2*, independently or in concert with rs35652421.

## 5. Conclusions

We have found a genotype association of rs35652124 with EO PD, a finding which supports the involvement of* NRF2* in PD. Our results indicate that* NRF2* has a disease modifying effect rather than affecting disease risk, but more detailed studies of the* NRF2* gene in larger populations are warranted. Quantification of mRNA in human blood cells showed that* NRF2* expression in control subjects and that of PD patients were similar and that the promoter SNP rs35652124 does not have a consistent effect on mRNA levels. Thus far, there is sparse functional evidence available to illuminate the role of genetic variants in* NRF2* in PD. Therefore it is also important to expand the knowledge on the function of* NRF2* in pathophysiology in order to conclude on the importance of this gene in PD.

## Supplementary Material

Supplementary Tables 1–3 contains test statistics from the logistic regression, haplotype, and the age stratifyed analyses respecively. Exact numbers for the gene expression data represented in Figures 1 and 2 can be found in supplementary Table 4.

## Figures and Tables

**Table 1:** Demographics of the study population.

	Participants, *n*	Females, *n* (%)	Mean age at enrolment, years	Mean age at diagnosis, years	Heredity, *n* (%)
Controls	509	290 (57.3)	71^*⋆*^	NA	NA
PD	501	184 (36.7)	67.2	59.5	88 (28.5)^*⋆⋆*^

PD: Parkinson's disease; NA: not applicable; ^*⋆*^age was unknown for 95 blood donors; ^*⋆⋆*^estimation based on 319 individuals for which this information was known.

**Table 2:** Results from genotype and allele analysis.

	Controls% (*n*)	PD% (*n*)	*Χ* ^2^ (DF)	OR (95% CI)	*p* value
*rs35652124*					
AA	47.7 (230)	45.0 (215)	0.79 (2)		0.68
AG	43.6 (210)	45.4 (217)			
GG	8.7 (42)	9.6 (46)			
A	69.5 (670)	67.7 (647)		1.09 (0.90–1.32)	0.40
G	30.5 (294)	32.3 (309)			

*rs6706649*					
GG	80.0 (387)	80.2 (385)	0.011 (2)		0.99
GA	18.6 (90)	18.3 (88)			
AA	1.4 (7)	1.5 (7)			
G	89.3 (864)	89.4 (858)		0.99 (0.74–1.32)	0.94
A	10.7 (104)	10.6 (102)			

*rs6721961*					
CC	74.8 (365)	77.4 (370)	2.39 (2)		0.30
CA	24.6 (120)	21.3 (102)			
AA	0.6 (3)	1.3 (6)			
C	87.1 (850)	88.1 (842)		0.91 (0.70–1.20)	0.54
A	12.9 (126)	11.9 (114)			

*rs2001350*					
AA	80.5 (388)	79.5 (387)	2.66 (2)		0.26
AG	19.3 (93)	19.5 (95)			
GG	0.2 (1)	1.0 (5)			
A	90.1 (869)	89.2 (869)		1.12 (0.82–1.48)	0.55
G	9.9 (95)	10.8 (105)			

*n*: number of individuals; PD: Parkinson's disease; *Χ*^2^: chi square; DF: degrees of freedom; OR: odds ratio; 95% CI: 95% confidence interval.

**Table 3:** Results from age stratified genotype and allele analysis for rs35652124.

rs35652124	Controls% (*n*)	LO PD% (*n*)	EO PD% (*n*)	*Χ* ^2^ (DF)	*p* value
AA	47.7 (230)	41.9 (163)	58.4 (52)	14.19 (4)	0.0067
AG	43.6 (210)	49.4 (192)	28.1 (25)		
GG	8.7 (42)	8.7 (34)	13.5 (12)		
A	69.5 (670)	66.6 (518)	72.5 (129)	3.82 (2)	0.15
G	30.5 (294)	33.4 (260)	27.5 (49)		

*n*: number of individuals; PD: Parkinson's disease; LO: late onset; EO: early onset; *Χ*^2^: chi square; DF: degrees of freedom.
