# Supplementary material for: Genetic Variations and mRNA Expression of NRF2 in Parkinson's Disease
Source: Parkinsons Dis. 2017 May 2;2017:4020198. doi: 10.1155/2017/4020198 (PMC5433415; doi:10.1155/2017/4020198)
Supplement: Supplementary file 1 — Supplementary Tables 1–3 contains test statistics from the logistic regression, haplotype, and the age stratifyed analyses respecively. Exact numbers for the gene expression data represented in Figures 1 and 2 can be found in supplementary Table 4. [file 4020198.f1.docx]

**Supplementary Tables**

**Supplementary table 1: Results from the logistic regression run under a genotypic model (2 degrees of freedom).**

| **SNP** | **minor allele** | **number of individuals** | **t-statistic coefficient** | **p-value** |
| --- | --- | --- | --- | --- |
| **rs35652124** | G | 958 | 1.124 | 0.5701 |
| **rs6706649** | A | 962 | 0.1191 | 0.9422 |
| **rs6721961** | A | 966 | 3.113 | 0.2109 |
| **rs2001350** | G | 967 | 2.729 | 0.2555 |

*SNP: single nucleotide polymorphism*

**Supplementary table 2: Results from the haplotype analysis**

| **haplotype*** | **frequency in controls** | **frequency in cases** | **Χ^2^** | **p-value** |
| --- | --- | --- | --- | --- |
| **AAAA** | 0.458 | 0.444 | 0.335 | 0.5626 |
| **AAAC** | 0.306 | 0.324 | 0.694 | 0.4048 |
| **AACA** | 0.106 | 0.107 | 0.005 | 0.9459 |
| **CCAA** | 0.095 | 0.099 | 0.071 | 0.7901 |
| **ACAA** | 0.026 | 0.019 | 1.055 | 0.3043 |

**single nucleotide polymorphisms are represented in the following order: rs2001350-rs35652124-rs6706649-rs6721961, Χ^2^: Chi-square*

**Supplementary table 3: Results from age stratified genotype and allele analyses**

| **rs6706649** | **Controls % (n)** | **LO PD % (n)** | **EO PD % (n)** | **X^2^ (df)** | **p-value** |
| --- | --- | --- | --- | --- | --- |
| **GG** | 80.0 (387) | 81.6 (319) | 74.2 (66) | 3.01 (4) | 0.56 |
| **GA** | 18.6 (90) | 16.9 (66) | 24.7 (22) |  |  |
| **AA** | 1.4 (7) | 1.5 (6) | 1.1 (1) |  |  |
| **G** | 89.3 (864) | 90.0 (704) | 86.5 (154) | 1.93 (2) | 0.38 |
| **A** | 10.7 (104) | 10.0 (78) | 13.5 (24) |  |  |
| **rs6721961** | |  |  |  |  |
| **CC** | 74.8 (365) | 77.7 (304) | 75.9 (66) | 2.56 (4) | 0.63 |
| **CA** | 24.6 (120) | 21.0 (82) | 23.0 (20) |  |  |
| **AA** | 0.6 (3) | 1.3 (5) | 1.1 (1) |  |  |
| **C** | 87.1 (850) | 88.2 (690) | 87.4 (152) | 0.53 (2) | 0.77 |
| **A** | 12.9 (126) | 11.8 (92) | 12.6 (22) |  |  |
| **rs2001350** | |  |  |  |  |
| **AA** | 80.5 (388) | 78.7 (314) | 83.0 (73) | 4.99 (4) | 0.29 |
| **AG** | 19.3 (93) | 20.1 (80) | 17.0 (15) |  |  |
| **GG** | 0.2 (1) | 1.3 (5) | 0 |  |  |
| **A** | 90.1 (869) | 88.7 (708) | 91.5 (161) | 1.63 (2) | 0.44 |
| **G** | 9.9 (95) | 11.3 (90) | 8.5 (15) |  |  |

*n; number of individuals PD; Parkinson’s Disease, LO; Late onset, EO; Early onset, X^2^; Chi square, df; degrees of freedom*

**Supplementary table 4: Relative *NRF2* mRNA levels in cases and controls**

| **status** | **age** | **gender** | **rs35652124** | **NRF2 expression*** | **SEM** |
| --- | --- | --- | --- | --- | --- |
| Control | 68 | F | AA | 0.57 | 0.14 |
| Control | 73 | M | AA | 1.08 | 0.14 |
| Control | 68 | F | AA | 0.98 | 0.14 |
| Control | 64 | F | AA | 0.58 | 0.13 |
| Control | 64 | F | AA | 0.86 | 0.13 |
| Control | 61 | F | AA | 4.05 | 0.43 |
| Control | 66 | F | AA | 1.12 | 0.15 |
| Control | 83 | M | AG | 3.61 | 0.28 |
| Control | 62 | F | AG | 0.93 | 0.07 |
| EO PD | 40 | M | AA | 0.63 | 0.23 |
| EO PD | 56 | M | AA | 1.22 | 0.16 |
| EO PD | 45 | F | AA | 1.03 | 0.19 |
| EO PD | 69 | M | AA | 0.18 | 0.05 |
| EO PD | 46 | M | AA | 1.81 | 0.21 |
| EO PD | 45 | M | AG | 4.62 | 0.68 |
| EO PD | 48 | F | AG | 0.57 | 0.09 |
| LO PD | 78 | M | AA | 1.06 | 0.43 |
| LO PD | 75 | F | AA | 2.59 | 0.62 |
| LO PD | 76 | M | AA | 1.76 | 0.27 |
| LO PD | 77 | M | AG | 1.27 | 0.32 |
| LO PD | 77 | F | AG | 0.66 | 0.16 |
| LO PD | 79 | F | AG | 1.52 | 0.27 |
| LO PD | 76 | F | AG | 1.07 | 0.33 |
| LO PD | 72 | M | GG | 6.76 | 1.00 |

*EO PD: early onset Parkinson’s disease, LO PD: late onset Parkinson’s disease, F: female, M: male, *mRNA levels were normalized to GAPDH and to a reference sample composed by cDNA from all control individuals, SEM: standard error of mean*
